# Arthroscopic-assisted retrograde mosaicplasty for an osteochondral defect of the femoral head without performing surgical hip dislocation

**DOI:** 10.1051/sicotj/2017030

**Published:** 2017-06-02

**Authors:** Onur Kocadal, Budak Akman, Melih Güven, Uğur Şaylı

**Affiliations:** 1 Department of Orthopaedics and Traumatology, Faculty of Medicine, Yeditepe University 34854 Istanbul Turkey

**Keywords:** Osteochondral defect, Osteochondral autograft transplantation, Mosaicplasty, Femoral head, Hip arthroscopy

## Abstract

Osteochondral autograft transplantation (mosaicplasty) for the osteochondral defects of the femoral head is generally performed with safe dislocation of the hip joint. Here, we present a 27-year-old male patient who had a symptomatic osteochondral defect of the femoral head and underwent mosaicplasty applied by hip arthroscopy without surgical hip dislocation. The preoperative radiological evaluation showed a focal osteochondral defect on the weight-bearing surface of the femoral head. With the assistance of hip arthroscopy, the defect area was reconstructed with osteochondral plug, which was taken from the ipsilateral knee, inserted in a retrograde fashion, and positioned with the spherical surface of the femoral head cartilage under fluoroscopic and arthroscopic control. At the final follow-up (26 months postoperatively), he had full range of motion (ROM) without pain and radiographic evaluation showed near complete incorporation of the graft with preservation of the native joint space. Mosaicplasty can be applied in a retrograde fashion with the assistance of hip arthroscopy without using surgical hip dislocation and may be an alternative treatment option for the osteochondral defects of the femoral head in young patients.

## Introduction

Osteochondral defects of the femoral head are rare lesions and their management is difficult due to complex anatomy of the hip joint. If left untreated, they are at risk of rapid progression to symptomatic arthritis that necessitates hip arthroplasty [1, 2]. Conservative treatment options are limited due to the low recovery capacity of cartilaginous tissue [1]. Surgical treatment options include arthroscopic lavage and debridement, chondroplasty or abrasion arthroplasty, microfracture, autologous chondrocyte implantation, osteochondral autograft or allograft transplantations, osteotomy, and limited resurfacing of the femoral head [1–7]. Irrespective of the method preferred, the aim of any cartilage repair technique should be restoration of articular surface congruity with preservation of normal joint kinematics [3].

Restoration procedure by using osteochondral autograft transplantation (mosaicplasty) is a well-established method, which involves the transfer of small bone plugs covered with healthy articular cartilage into focal chondral defects. Mosaicplasty technique has been well described in the literature for osteochondral defects locating in the knee and ankle joints [8]. However, utilization of the same technique for the hip joint is limited and technically demanding. Patients with osteochondral defects in the hip joint generally undergo surgical hip dislocation with or without a trochanteric slide osteotomy for placement of the osteochondral plug [2, 4, 6, 8, 9].

Advances in hip arthroscopy have broadened the spectrum of tools available for diagnosis and management of hip pathologies [1, 10–12]. However, the literature is still not sufficient to draw firm conclusions regarding the usage of hip arthroscopy for osteochondral defects. We present a young male patient who had a symptomatic osteochondral defect of the femoral head and underwent subsequent treatment using an osteochondral autograft transplantation applied in a retrograde fashion via a hip arthroscopy without surgical hip dislocation.

## Case report

A healthy 27-year-old male patient was admitted to the first author’s institution with left hip pain for 10 months of duration without a history of a distinct trauma. He had severe pain in the left groin on weight bearing, with variable degree of pain at rest. He also expressed some mechanical symptoms of the affected hip, including catching and locking related to work and daily living activities. He did not have any systematic disease nor any congenital or developmental hip diseases. He did weekly jumping and running activities including basketball and soccer. On clinical examination, he had an antalgic gait and ambulated with a cane. Although the range of motion (ROM) of the hip joint was not restricted, his hip was painful with each of the extremes of passive flexion, internal rotation, and abduction. There was no compensatory external rotation with hip flexion. On the first evaluation, the Harris hip score of his left hip was 62 points. He did not receive any conservative treatment before the admission.

**Figure 1.**
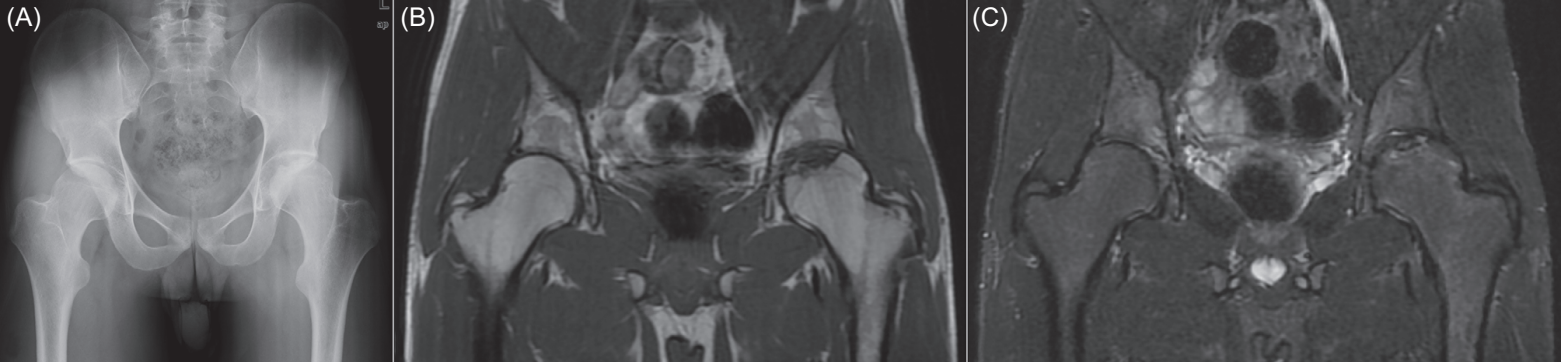
Pelvis anteroposterior radiograph (A) reveals an osteochondral defect on the weight-bearing surface of the femoral head. Coronal T1 (B) and T2 (C) weighted MRI images show the osteochondral lesion without any displacement or evidence of collapse.

The anteroposterior radiograph of the pelvis showed a focal osteochondral defect on the weight-bearing surface of the femoral head (Figure 1A). The subchondral lesion was clearly seen separate and isolated from the remainder of the femoral head by a radiolucent line. Lateral center edge, sharp, femoral neck shaft, alpha angles, and acetabular inclination were 38°, 40°, 131°, 44.9°, and 44.3°, respectively. All these radiological parameters were within normal limits. Magnetic resonance imaging (MRI) scans showed an osteochondral separation without any displacement and evidence of collapse of the major fragment (Figures 1B and 1C).

**Figure 2.**
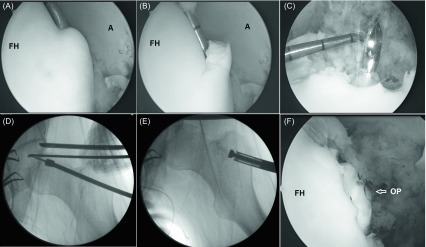
(A, B) Arthroscopic views of the osteochondral defect in the superior dome of the femoral head. The anterior portal was used for instrumentation and the anterolateral portal was used for viewing. After excision of the chondral defect, the tip of the K wire, which is passed from the lateral border of the femur below the trochanter major, is visualized with an arthroscope (C). Drilling is performed over the K wire with a 10 mm diameter drill (D). The osteochondral plug is transferred to the femoral head in a retrograde fashion (E) and positioned in the spherical surface of the femoral head cartilage (F). (FH: Femoral head, A: Acetabulum, OP: Osteochondral plug).

Hip joint arthroscopy was recommended to the patient. Under general anesthesia, a left hip arthroscopy was performed in a usual manner while the patient lay in a supine position on a fracture table. The standard anterolateral portal was used to visualize the hip joint with a 70° arthroscope and the anterior portal was used for instrumentation. The anterior hip joint capsule was released via the anterior portal and the joint space was identified clearly. There was no acetabular labral tear or distinct femoroacetabular impingement. A round osteochondral defect of 10 mm diameter was identified at the superior dome of the femoral head (Figures 2A and 2B). The chondral lesion was excised and the defect was curetted and shaved. Under fluoroscopic control, a K wire with a 1.5 mm diameter was passed from the lateral border of the femur below the trochanter major and the tip of the K wire was visualized with an arthroscope (Figure 2C). Then overdrilling was performed over the K wire with a 10 mm diameter drill to prepare the host defect (Figure 2D). The osteochondral cylindrical plug with a 10 mm diameter and 15 mm in length was harvested from the non-weight-bearing area of the proximal lateral femoral condyle of the ipsilateral knee through a small lateral parapatellar arthrotomy using the Osteochondral Autograft Transfer System (OATS) (Arthrex, Naples, Florida, USA). The harvested osteochondral plug was transferred to the femoral head in a retrograde fashion and positioned within the spherical surface of the femoral head cartilage using fluoroscopic and arthroscopic control until the plug and intact chondral articulation were level (Figures 2E and 2F). Afterward, the femoral tunnel was filled with a 60 mL spongious allograft. The arthroscopic portals and the incision on the knee joint were closed in a standard fashion.

**Figure 3.**
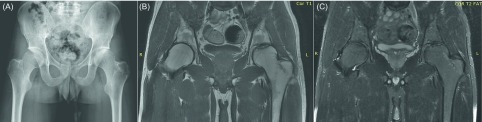
On the final follow-up (26 months postoperatively), the pelvic anteroposterior radiograph (A) shows near complete incorporation of the osteochondral plug with well-preserved joint congruency and joint space. Coronal T1 (B) and T2 (C) weighted MRI images confirm the stability of the plug and ongoing healing.

There were no peri- and postoperative complications related to the procedure. Active and passive hip ROM exercises were started on the second day postoperatively and he was discharged home the day after surgery. He was mobilized non-weight bearing with two crutches for six weeks. At the end of the sixth week, he gradually started weight bearing and at the end of the 10th week, he was allowed to walk with full weight bearing. At the final follow-up (26 months postoperatively), he had a full range of movement without any complaints of rest pain or pain related to activities. The Harris hip score of his left hip was 96 points. Postoperative radiographs at the final follow-up showed near complete incorporation of the graft with preservation of the native joint space (Figure 3A). MRI scans confirmed the stability of the osteochondral plug and ongoing healing (Figure 3B). Informed consent for publication was obtained from the patient.

## Discussion

Satisfactory clinical and radiographic results have been reported with mosaicplasty [2, 4, 6, 8, 9] or osteochondral allograft transplantation procedures [3, 5], which were performed for osteochondral defects of the femoral head via surgical hip dislocation. Girard et al. [4] reported the results of mosaicplasty through a trochanteric flap osteotomy with surgical hip dislocation. Ten patients under the age of 25 years were followed up for a mean period of 29.2 months. Hip scores improved and the ROM of the hip joints increased in all patients postoperatively. Radiographic investigations showed good incorporation of the autografts at the site of osteochondroplasty. Khanna et al. [5] reported the results of fresh osteochondral allograft for cartilage restoration of the femoral head by using the same surgical technique. They treated 17 patients with various etiologies. After a mean follow-up of 41.6 months, Harris hip scores improved significantly. Thirteen patients had fair to good results. However, in one patient, secondary surgery including a repeat allograft was required and three patients underwent hip replacement. Kılıçoğlu et al. [6] reported the long-term result of mosaicplasty in a 27-year-old male patient who had been treated for a femoral head cartilage defect with trochanteric flap osteotomy and surgical hip dislocation. After eight years of follow-up, the hip ROM of the patient was preserved and the Harris hip score was excellent with 96 points.

Despite these satisfactory results and the advantage of a wide exposure to allow visualisation of the entire femoral head, the mosaicplasty technique applied by surgical hip dislocation is an invasive procedure. On the other hand, hip arthroscopy as a minimally invasive technique has gained popularity in recent years and its indications have shifted from being a diagnostic tool to a therapeutic one [7]. The most common indication for hip arthroscopy is femoroacetabular impingement and associated labral pathologies. However, for chondral defects of the femoral head, hip arthroscopy is commonly used as a diagnostic method and/or a debridement procedure [7]. There are a few published series about arthroscopic treatment of osteochondral lesions of the hip joint. Field et al. [10] described an arthroscopic-assisted technique to graft chondral defects and subchondral cysts of the acetabular socket using a synthetic osteochondral plug. They treated four patients with solitary cysts in the roof of the acetabulum. A bone tunnel was prepared from the region of the iliac crest to the acetabular articular surface, and a synthetic osteochondral plug was inserted in an antegrade fashion and positioned flush with the lunate articular cartilage by using arthroscopic control. After a mean follow-up of 10 months, radiographic assessment confirmed the stability of the osteochondral plugs and all patients showed an improvement in symptoms and function. Kubo et al. reported a patient with follow-up of two years who was treated with arthroscopic antegrade osteochondral autologous transplantation technique for an osteochondral lesion of the femoral head [11]. The authors stated that this arthroscopic technique is a suitable method for osteochondral lesions located on the femoral head.

Arthroscopic-assisted mosaicplasty can also be considered 15 for patients with an osteochondral defect of the femoral head statement [12]. The arthroscopic technique used in the present report is a new concept being first described by Çetinkaya et al. [13]. They used a retrograde osteochondral autograft transplantation technique combined with hip arthroscopy in two patients. Radiographic investigations in both patients showed a focal osteochondral defect on the weight-bearing area of the femoral head and distinct femoroacetabular impingement. On the final follow-up (2 and 2.5 years postoperatively), both patients had significant improvement in the Harris hip score. They concluded that this technique yielded good short- and mid-term clinical and radiographic outcomes. There are some technical differences between the present case and that in their report. In both of their cases, the K wire was centered in the chondral defect by using an anterior cruciate ligament (ACL) guide. Although we did not use this guide, we believe that using an ACL guide can simplify the surgical technique. Under fluoroscopic control with an ACL guide, they preferred to drill the femoral head one size under that of the harvested plug, so as to constitute a press-fit. In the present case, we drilled the femoral head to the same size as the harvested plug. On the final follow-up (26 months postoperatively), the MRI scans confirmed the stability of the osteochondral plug and the patient had satisfactory functional and radiographic results. We believe that the arthroscopic retrograde mosaicplasty is a minimally invasive technique that allows early rehabilitation. This technique allows intervention for lesions such as labral tears associated with osteochondral pathologies. However, it should be taken into consideration that neurovascular and iatrogenic chondral injuries can occur during this surgery.

## Conclusion

Retrograde grafting technique described in the present report is much safer and minimally invasive, when compared with the safe dislocation method. This case suggests that mosaicplasty can be applied in a retrograde fashion via hip arthroscopy without performing surgical hip dislocation and it may be an alternative treatment option for osteochondral defect of the femoral head in young patients.

## Conflict of interest

The authors declare that they have no conflict of interest.
